# Antimicrobial Resistance of *Campylobacter* *jejuni*, *Escherichia coli* and *Enterococcus* *faecalis* Commensal Isolates from Laying Hen Farms in Spain

**DOI:** 10.3390/ani11051284

**Published:** 2021-04-29

**Authors:** Jorge Rivera-Gomis, Pedro Marín, Cristina Martínez-Conesa, Julio Otal, María José Jordán, Elisa Escudero, María José Cubero

**Affiliations:** 1 Research Group E095-06 Antimicrobial Resistance in Animal Health, Regional Campus of Excellence Mare Nostrum, University of Murcia, 30100 Espinardo, Murcia, Spain; pmarin@um.es (P.M.); escudero@um.es (E.E.); mjcubero@um.es (M.J.C.); 2 Research Group on Rainfed Agriculture for Rural Development, Department of Rural Development, Oenology and Sustainable Agriculture, Murcia Institute of Agri-Food Research and Development (IMIDA), 30150 Alberca de las Torres, Murcia, Spain; cristina.martinez4@carm.es (C.M.-C.); mariaj.jordan@carm.es (M.J.J.); 3 Animal Production Department, Regional Campus of Excellence Mare Nostrum, University of Murcia, 30100 Espinardo, Murcia, Spain; juotal@um.es

**Keywords:** critically important antibiotics, antimicrobial categories, minimum Inhibitory concentration, multidrug-resistance, public health risk

## Abstract

**Simple Summary:**

Antimicrobial resistance (AMR) is a global threat for human and animal health. Few studies have been carried out in laying hens. We evaluated the antimicrobial susceptibility of commensal *Campylobacter jejuni*, *Escherichia coli*, and *Enterococcus faecalis* isolates in Spanish laying hens in 2018. *C. jejuni* was highly resistant, and a medium proportion of the isolates were susceptible to all the antimicrobials studied. *E. coli* showed medium to high percentages of resistance to the antibiotic categories of highest public health risk concern (A and B). Only a low proportion of the isolates were susceptible to all antimicrobials. The *E. faecalis* resistance to antimicrobials was variable, and very few isolates were susceptible to all antimicrobials. Novel data on AMR in laying hen commensal isolates in Spain was provided, and the AMR levels differed from those reported for poultry in the EU. High resistance to key drugs used in human medicine was found. Therefore, laying hens could be a source of AMR for humans, thus, representing a public health risk.

**Abstract:**

Antimicrobial resistance (AMR) is a global threat for human and animal health. Few studies have been carried out in laying hens. We evaluated the antimicrobial susceptibility of commensal *Campylobacter jejuni*, *Escherichia coli*, and *Enterococcus faecalis* isolates in Spanish laying hens in 2018. The Minimum Inhibitory Concentration (MIC) was used to identify any AMR of the studied isolates by means of a broth microdilution method. *C. jejuni* was highly resistant to the B category antimicrobials, and 52% of the isolates were susceptible to all the antimicrobials tested. *E. coli* showed medium and high percentages of resistance to the B and A antibiotic categories, respectively, and 33.33% of the isolates were susceptible to all antimicrobials. The *E. faecalis* resistance to A category antimicrobials was variable, and 4.62% of the isolates were susceptible to all antimicrobials. In our work, novel data on AMR in laying hen commensal isolates in Spain is provided, and the AMR levels differ from those reported for poultry in the EU. A high resistance to key drugs for human medicine was found, representing a public health risk.

## 1. Introduction

Antimicrobial resistance (AMR) represents a serious public health concern, as infections caused by antibiotic-resistant bacteria are associated with significant morbidity and mortality worldwide in humans [1]. The effect of the clinical use of antibiotics in livestock production is a subject of debate [2]. However, the detection of the *mcr-1* colistin resistance gene in swine, imported chicken (in Denmark), and hospitalized patients has helped to settle the argument that antibiotics used in veterinary medicine can be a source of resistance genes for pathogenic bacteria to humans [3,4]. AMR is a complex ecological problem that threatens human, animal, and environmental health in a “One Health” context [5].

The poultry industry is one of the fastest-growing components of the livestock sector worldwide. The European Union (EU) is an important producer of poultry meat and eggs and has around 400 million laying hens in production. Spain accounts for 10% of the eggs produced in the EU [6]. Egg laying hens (layers) are kept in conditions according to EU legislation [7]. The egg production sector in Spain is widely integrated in large companies. Around 23% of the total census of Spanish layers are kept under alternative systems, while more than 85% are kept in enriched cages [8]. Antimicrobials are frequently used in poultry production [9,10]. 

The most common pathologies in laying hens treated with antibiotics are digestive, which are typically treated with colistin or erythromycin, and respiratory, which are treated with tylosin. The antibiotics used in commercial poultry can be divided into two categories depending on their use: therapeutic antibiotics and growth-promoting antibiotics [11]. In countries outside the EU, antibiotics used in feed for growth promotion and disease prevention purposes are administered at lower or subtherapeutic levels than antibiotics used for disease treatment. 

This use has been banned in the EU since 2006 [12]. In feed, antibiotics can cause a selection pressure on bacterial populations that leads to an increased AMR in human pathogens [9]. Human food safety concerns have favoured the EU ban on the use of antimicrobials as growth promoters in food production and the increase of AMR surveillance in food-borne pathogens and indicator organisms. 

According to the European Medicines Agency (EMA), antibiotics are classified according to the potential consequences for public health of increased AMR due to their use in animals and depending on the necessity to use them in veterinary medicine. In the EMA categorisation, there are four categories, A (Avoid), B (Restrict), C (Caution), and D (Prudence), with the latter as the lowest risk category [13]. Antibiotics from the A category are reserved for human treatment only, and their use is not allowed in food-producing animals. B category antibiotics are critically important antibiotics, and should be used only as a last option after susceptibility testing has been conducted when no other antibiotic would be clinically effective. These antibiotics are also considered critical by the WHO [14].

Standardized and continuous surveillance programmes are necessary to monitor the occurrence and persistence of AMR in food animals [1,15]. Indicator bacteria are generally used to monitor antimicrobial resistance since they can be commonly found in healthy animals. In addition, these bacteria acquire antimicrobial resistance faster than other commonly found bacteria [15,16]. AMR in poultry is monitored through the study of indicator commensal bacteria. *Campylobacter* spp., *Escherichia coli*, and *Enterococcus* spp. are frequently used as such indicators due to their frequent presence in the intestines of birds [17].

Campylobacteriosis has been the most commonly reported zoonotic disease in the EU since 2005 [18]. Domestic poultry are the main reservoir of these microorganisms in the absence of clinical signs [19]. AMR in *Campylobacter* spp. represents a serious public health concern due to the increasing number of *Campylobacter* strains resistant to several drugs that can be isolated from human samples, as well as from animals and food [20].

*E. coli* is usually present in the avian digestive system, and it can contaminate eggs during laying [21]. Avian extraintestinal pathogenic *E. coli* can be transmitted through food, thus, causing disease in humans worldwide, and particularly affecting immunocompromised people [22]. There is significant evidence of AMR in *E. coli* isolated from commercially raised chickens [23].

Enterococci are emergent pathogens and certain species, such as *Enterococcus faecalis* and *E. faecium*, cause opportunistic nosocomial infections in humans. Food animals act as reservoirs of antibiotic-resistant enterococci due to the use of antimicrobials prophylactically or as growth promoters [24]. 

The omnipresence of poultry meat and eggs in the human diet and the related antimicrobial use to this food-producing sector have suggested the poultry industry as a source of AMR bacteria pathogenic to humans. Animal foods, including eggs, play an important role regarding the transmission of AMR bacteria and genetic material to humans. Nevertheless, the research on resistance profiles of commensal bacteria present in laying hens farms are scarce. 

In a previous study on commensal *Campylobacter* spp., *E. coli* and *Enterococcus* spp. in Spanish laying hens, worrisome levels of resistance to the C and D categories of antimicrobials were found, particularly regarding the antimicrobials used in human medicine [25]. The present study investigated AMR and multidrug-resistance regarding A and B antibiotic categories in commensal *Campylobacter jejuni*, *E. coli,* and *E. faecalis* strains isolated in 2018 from laying hens farms in Spain.

## 2. Materials and Methods

### 2.1. Sampling 

**Figure 1:**
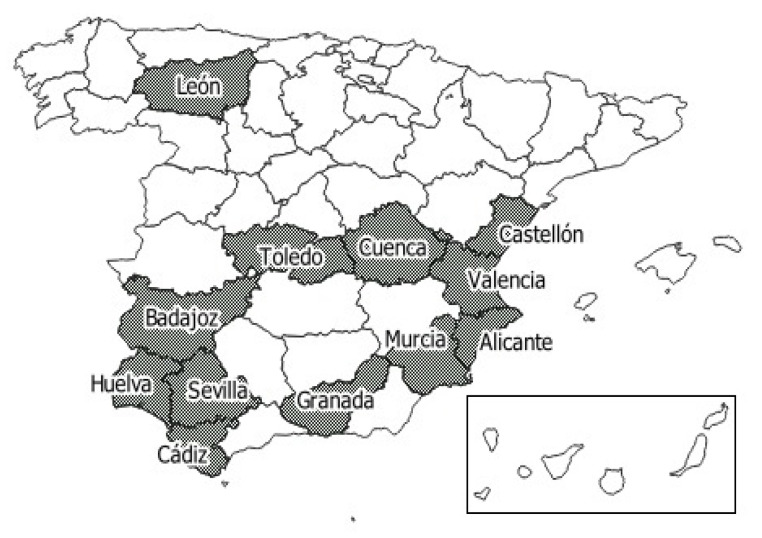
The Spanish provinces where the sampled farms were located.

Samples were collected from 39 laying hen farms, between April and November 2018, located in 12 provinces of the six Spanish regions that produce 62% of Spanish eggs [8] (Figure 1). The breeding, biosecurity, and biosafety practices and protocols were similar between the studied farms. Sampling was carried out during the 40 and 50 laying weeks [26]. All the farms that participated in the study used cage systems to keep the hens.

A total of 39 samples of 250 g of faeces (one per farm) were taken. The sampling was distributed in 10 random sampling points of the floor where the layers were kept. Sterile plastic containers were used for the collection of the samples. Transport to the laboratory was performed under refrigeration conditions by courier services. A cold chain was maintained, and the samples were processed within 24 h of their arrival at the laboratory.

### 2.2. Isolation and Molecular Identification of C. jejuni, E. coli, and E. faecalis Commensal Strains

*C. jejuni* commensal strains were isolated as indicated in ISO 10272-1:2006. The faecal samples were diluted in peptone water (1:10) and inoculated in modified Charcoal Cefoperazone Deoxycholate Agar (mCCDA, Oxoid, UK) plates. Incubation was done at 42 °C for 48 h in a micro aerobic environment (5% O_2_, 10% CO_2_, 85% N_2_) [27] that was generated with Campy Gen (Oxoid, UK). Simplex PCR was used for the genus and species identification of three microscopically confirmed *Campylobacter* isolates per sample. The primer sequence and the cyclic conditions used were those described by Linton et al. [28] and Nayak et al. [29] for the *Campylobacter* genus and species, respectively.

A peptone water dilution (1:10) of the samples was carried out. The culture medium Rapid’ E. Coli2 (Bio-Rad) was used to isolate the *E. coli* strains (ISO 16649-2). A simplex PCR assay was used for genus confirmation in three isolated strains per sample (ISO 22174: 2005) [25].

KF streptococcus agar (Thermo Scientific™ CM0701B) medium was used for the isolation of *E. faecalis*, which was confirmed by PCR [30]. Brain heart infusion broth (Bio-Rad) with 20% glycerol was used to store five PCR confirmed isolates from each sample at −80 °C for later analysis. A total of 195 *E. coli*, 195 *E. faecalis,* and 25 *C. jejuni* isolates were studied. The samples were distributed across Spain, originating from 6 of the 17 regions for *E. coli* and *E. faecalis* and from 4 regions for *C. jejuni* [25].

### 2.3. Antimicrobial Susceptibility Testing

A growth suspension was prepared in Tryptic soy broth from a 24-h culture and adjusted to a turbidity of 0.5 McFarland standard. This was inoculated on Muller–Hinton broth and incubated at 37 °C for 24 h. Cation-adjusted Mueller–Hinton broth supplemented with 2.5–5% lysed horse blood was used for *C. jejuni*, with incubation at 37 °C for 24 h in a microaerobic atmosphere [31].

The A category and B category antimicrobials used in the study are indicated in Table 1. The quality control organisms used were *C. jejuni* (ATCC 33560), *E. coli* (ATCC 25922), and *E. faecalis* (ATCC 29212).

The Minimum Inhibitory Concentration (MIC) was used to identify AMR of the studied isolates by means of a broth microdilution method using the SensitreTM system (Thermo Fisher). The guidelines of the European Committee on Antimicrobial Susceptibility Testing were used to interpret the results [17,32,33].

The MIC_50_ and MIC_90_ for each antibiotic were considered as the MICs at which 50% and 90% of the isolates were inhibited, respectively. Multidrug-resistant (MDR) isolates were considered as such when phenotypic resistance to three or more antimicrobial classes was detected [34]. The epidemiological cut-off value (ECOFF) of the broth microdilution phenotypic test was considered as the separation between the susceptible wild-type bacterial population from non-wild-type isolates that had a reduced susceptibility to an antimicrobial agent [35]. We used the ECOFF values indicated in EU legislation [17].

### 2.4. Data Analysis

SPSS software (version 16) was used to generate frequency and proportion values of the antimicrobial resistance profiles from the data collected.

## 3. Results

### 3.1. MIC Distributions

**Figure 2:**
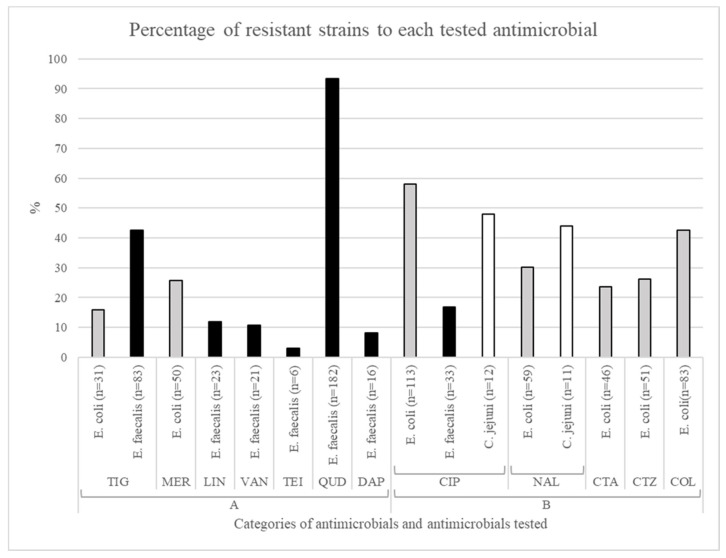
Distribution of resistant strains for the tested antimicrobials in the investigated microorganisms.

The percentages of resistance for the 25 *C. jejuni*, 195 *E. coli* and 195 *E. faecalis* isolated strains were categorized as very low (0–1%), low (>1–10%), medium (>10–50%), high (>50–70%), and very high (>70%) [32]. The MIC distributions of the antimicrobials tested against *C. jejuni* are summarized in Table 2. The tested strains showed a relatively medium proportion of resistance to ciprofloxacin and nalidixic acid (48% and 44% respectively) (Figure 2).

The MIC distributions for *E. coli* were defined (Table 3). Resistance to B category antibiotics was found in medium to high percentages of the tested strains (cefotaxime 23.59%, ceftazidime 26.15%, nalidixic acid 30.26%, colistin 42.56%, and ciprofloxacin 57.95%). Medium percentages of resistant strains were found for meropenem (25.64%) and for tigecycline (15.90%), which are both included in the A category (Figure 2).

The MIC distributions of the antimicrobials tested against *E. faecalis* are summarized in Table 4. The resistance to A category antibiotics was variable, ranging from low (teicoplanin 3.08%) and medium (vancomycin 10.77%, linezolid 11.79%, tigecycline 42.56%) to a very high resistance levels to quinupristin-dalfopristin, with a 93.33% of resistant strains. A medium proportion of strains were resistant to ciprofloxacin (16.92%), which is included in the B category (Figure 2).

### 3.2. Antimicrobial Resistance Pattern to Antimicrobials of Classes A and B 

From the 25 *C. jejuni* strains, 52% were susceptible to all the antimicrobials tested, and 48% were resistant to quinolones (B category), which was the only antimicrobial family tested.

Among the 195 *E. coli* isolates, 33.33% were susceptible to all antimicrobials tested. There was no multidrug-resistance to all the tested families of antimicrobials (Table 5). A 13.33% of the isolates showed partial profiles of multidrug-resistance to three families of antimicrobials, and 17.95% were MDR to four families (considering that only antimicrobials from A and B categories were tested). The most frequently observed *E. coli* partial resistance profiles corresponded to: (a) quinolones (B category) (15.38%) and (b) carbapenems, cephalosporins, and polymyxins (A category) and quinolones (B category) (15.38%).

A total of 4.62% of the 195 *E. faecalis* isolates showed susceptibility to all antimicrobials. MDR to all the studied antimicrobial classes was not found (Table 5). MDR to three antimicrobials was found in 9.74% of the isolates (Table 5). The most frequent *E. faecalis* resistance profiles were: (a) streptogramins (A category) (36.92%) and (b) streptogramins and glycilcyclines (A category) (24.10%).

## 4. Discussion

This study focused on AMR to the A and B categories of antimicrobials in commensal bacteria present in healthy Spanish laying hens in 2018. The levels and profiles of resistance were identified for commensal *C. jejuni*, *E. coli,* and *E. faecalis* and isolates. Data on the antimicrobial susceptibility obtained in this work can be an indicator of AMR and antimicrobial use in laying hen farms in Spain. Another source of antimicrobial resistance that should be further studied in laying hens, in addition to antimicrobial use, is the vertical transmission of resistance genes. 

This could partially explain some of the observed AMR prevalence in this productive sector despite the reduced antimicrobial treatments [36]. This has been previously identified in poultry regarding fluoroquinolone resistance in the absence of fluoroquinolone use [37]. The differences in the AMR values observed in this study in laying hens with respect to broilers could be related to the specific characteristics of the productive systems, such as the use of different antimicrobials for different frequent pathologies, the longer life cycle of hens, and the way that the animals are raised (layers are predominantly in cages, and broilers are on the floor).

A 48% of resistance to ciprofloxacin and a 44% resistance to nalidixic acid (both from B category) were found for *C. jejuni*. These values of resistance were much lower than those found for ciprofloxacin for the same bacteria in broilers from Spain (87.26%) and the EU (73.54%) [18]. Ciprofloxacin is a critically important antibiotic to treat human campylobacteriosis [14,38]. The percentage of resistance to ciprofloxacin found in laying hens (48%) was lower than those found in humans for *C. jejuni* in Spain (90.1%) and EU (59.3%) [18]. The resistance to nalidixic acid in *C. jejuni* isolated from laying hens (44%) was much lower than the values reported in broilers by the European Food Safety Authority (EFSA) and the European Center for Disease Prevention and Control (ECDC) of 86.62% in Spain and 70.53% in the EU [18].

Regarding the *E. coli* findings for AMR to B category antimicrobials, the value found for AMR to ciprofloxacin in laying hens (57.95%) was lower than that for Spanish broilers in 2018 (80.00%) but similar to the EU value (55.89%). Nalidixic acid resistance values were 30.26% for Spanish laying hens, while the values were much higher for Spanish (75.88%) and European broilers (51.00%) [18]. Colistin (B category) is considered a critically important antimicrobial due to its use as a drug of last resort in human medicine against MDR Gram-negative bacteria [14]. 

A large difference was found between colistin resistance in Spanish laying hens (42.56%) compared to the values reported for broilers in Spain (0.00%) and in the EU (0.70%). The resistance levels found in Spanish laying hens to ceftazidime (26.15%) and cefotaxime (23.59%) (B category), although moderate, were much higher than those reported by EFSA and ECDC [18] in 2018 for broilers, being 3.53% for ceftazidime and 3.53% for cefotaxime in Spain and 2.79% for ceftazidime and 3.00% for cefotaxime in the EU. 

Regarding A category AMR for *E. coli* in laying hens, there were large differences between the values reported at the national and European level for poultry and the findings of our study. A 25.64% of tested strains were resistant to meropenem and 15.90% were resistant to tigecycline, while those values were 0.00% at the Spanish and 0.05% at the EU level in broilers. These antibiotics are considered high priority antimicrobials by the WHO [14].

Regarding the total susceptibility, 33.33% of the 195 *E. coli* studied strains were susceptible to all antimicrobials tested from the A and B categories. The total susceptibility to antimicrobials from all classes in broilers was 7.06% in Spain and 22.88% in the EU [18]. In Spanish laying hens, the MDR to four families of antibiotics was found in 17.95% of the isolates. Multidrug-resistance in broilers, considering all antimicrobials, was 46.87% at the EU level and 49.41% in Spain. 

The multidrug-resistance profiles in this work were significantly different than those reported for Spain and the EU by EFSA and ECDC [18] due to the presence of meropenem (carbapenem) combined with cephalosporins, polymyxins (A category), and quinolones (B category), which was the most frequent MDR profile observed in this research in 15.38% of the *E. coli* isolates. Due to the limited number of antibiotics included in the present study, the number of MDR profiles that could be identified was restricted.

In European poultry, there are limited data on AMR for *Enterococcus* spp. This study provides a clear overview of AMR of commensal *E. faecalis* isolated from laying hens in Spain. The obtained percentage of resistance to vancomycin was medium (10.77%); however, this was higher than what was reported in broilers in the EU, where vancomycin resistant enterococcus (VRE) (*E. faecium* and *E. faecalis*) were not found or presented a low prevalence from 2004 to 2014 in the EU [39]. The percentage of commensal VRE has been decreasing in farm animals and in humans since 1997, when the use of avoparcin in food-producing animals was forbidden in the EU [40,41]. 

The low occurrence of VRE in chickens [39] contrasts with the situation in enterococci from laying hens (our study) and human clinical samples [42]. Values for vancomycin resistant *E. faecalis* were found to be 1.1% [43] at the European level in human medicine [42]. The value of resistance to vancomycin in *E. faecalis* found in laying hens was higher than in other animal productions and should be further investigated, as this could represent a source of AMR to this antibiotic. The highest percentage of resistance obtained was for quinupristin-dalfopristin (93.33%), which was expected considering that *E. faecalis* are intrinsically resistant to this antibiotic [44]. This antibiotic is very important in hospital intensive care units to treat vancomycin-resistant *E. faecium* infections [45,46].

For tigecycline, the percentage of resistant *E. faecalis* reached a medium value (42.56%) in laying hens. This was much higher than the reported levels in broilers in the EU, where tigecycline-resistant enterococcus (*E. faecium* and *E. faecalis*) were not present from 2008 to 2014 [39]. Tigecycline, a glycylcycline, which is approved and used for several clinical applications in human medicine, is a minocycline derivative capable of overcoming several tetracycline resistance mechanisms [47]. These high levels of AMR found in laying hens are likely related to the inappropriate use of different tetracyclines in poultry. 

Lower percentages were obtained for linezolid (11.79%), ciprofloxacin (16.92%), daptomycin (8.21%), and teicoplanin (3.08%). These percentages are not less worrying, because they are antibiotics from the A and B categories, which are reserved for human treatment or as a last resort when no other antibiotic would be clinically effective. Enterococci are growing in importance as nosocomial infective agents. This fact, together with the high AMR levels to antimicrobials required in human medicine, poses a great concern. In addition, the flow of resistance-associated genetic material between poultry and human enterococci and the transmission of resistant enterococci from animals to humans have been proven [48,49].

The antibiotics used to treat pathologies in laying hens, such as colistin, erythromycin, and tylosin, could potentially favour the development of AMR in both commensal and pathogenic bacteria, also increasing the risk of AMR transfer. Faecal shedding by carrier animals is an important source of AMR bacterial contamination of meat and poultry products [50] and may also be responsible for contamination of fruit and vegetables through the environment [51]. *Campylobacter* spp. are a very common cause of food and waterborne infection in many countries, and their resistance to antimicrobials has been linked with the greater severity and longer duration of infections [52,53]. Regarding *E. coli*, poultry has been identified as an important source of resistant strains of these bacteria for humans [54,55,56].

## 5. Conclusions

High levels of AMR to the A and B EMA categories of antimicrobials [13] were found in Spanish laying hen farms. Farmers and veterinarians should consider these AMR levels and pay special attention to the antibiotics and doses used when treating laying hens. The antimicrobials used in laying hens typically have no withdrawal period. In cases where there is a withdrawal period, the eggs are destroyed. 

These results are important from a public health perspective, as laying hens were identified as a source of AMR bacteria that could transfer resistance to human pathogens, such as *Campylobacter* spp. and *E. coli*. The results obtained in this research regarding AMR to the antibiotics used in human medicine are even more worrisome when we consider that, in a previous work, high levels of AMR to antibiotics from the C and D categories used in human medicine were found in *Campylobacter* spp., *E. coli*, and *Enterococcus* spp. [25]. 

Further research is needed on the resistance to antimicrobials in laying hens in the EU. The risk of laying hen farms related AMR transmission to humans due to direct contact, environmental dissemination, and egg consumption should be further investigated. In several cases, the data on AMR reported for poultry differed from what we found in laying hens, especially regarding antimicrobials that are important for human medicine, such as ciprofloxacin resistance in *Campylobacter* spp., colistin, meropenem, and tigecycline in *E. coli* or quinupristin-dalfopristin and tigecycline in *Enterococcus* spp. 

Therefore, a better representation of the laying hen sector regarding AMR monitoring is required in order to have clear information on this issue, which is similar to other animal production sectors [18]. This information is essential to implement actions according to the real AMR situation in the sector. Finally, programmes designed to reduce antibiotic consumption and tackle AMR in food-producing animals, such as the Spanish PRAN programme [57], should include laying hens considering the high AMR levels found in this sector as found in this research.

## Figures and Tables

**Table 1:** Antimicrobials used in the study.

Microorganism	A Category Antimicrobials	B Category Antimicrobials
*C. jejuni*		Quinolones (ciprofloxacin, CIP; nalidixic acid, NAL)
*E. coli*	Glycylcyclines (tigecycline, TIG) Carbapenems (meropenem, MER)	Cephalosporins (cefotaxime, CTA; ceftazidime, CTZ) Polymyxins (colistin, COL) Quinolones (ciprofloxacin, CIP; nalidixic acid, NAL)
*E. faecalis*	Glycylcyclines (tigecycline, TIG) Glycopeptides (vancomycin, VAN, teicoplanin, TEI) Lipopeptides (daptomycin, DAP) Oxazolidinones (linezolid, LIN) Streptogramins (Quinupristin-dalfopristin, QUD)	Quinolones (ciprofloxacin, CIP)

**Table 2:** Minimum Inhibitory Concentration (MIC) distributions of antimicrobials against 25 *C. jejuni* strains.

EMA Category	Antimicrobial Agent	Distribution of MICs (in µg/mL and Number of Strains)																			MIC_50_	MIC_90_	ECOFF
		0.0075	0.015	0.03	0.064	0.125	0.25	0.5	1	2	4	8	16	32	64	128	256	512	1024	2048			
B Category	Ciprofloxacyn	0	0	0	**52**	**0**	**0**	**0**	**4**	**4**	**8**	**20**	**12**	0	0	0	0	0	0	0	≤0.125	≥16	>0.5
	N° Strains				13				1	1	2	5	3										
	Nalidixic acid	0	0	0	0	0	0	**56**	**0**	**0**	**0**	**0**	**0**	**4**	**40**	0	0	0	0	0	≤1	≥64	>16
	N° Strains							14						1	10								

The vertical bars indicate the epidemiological cut-off value (ECOFF) value for each antibiotic [17]; The bold numbers indicate the percentage of strains for every MIC value.

**Table 3:** Minimum Inhibitory Concentration (MIC) distributions of antimicrobials against 195 *E. coli* strains.

EMA Category	Antimicrobial Agent	Distribution of MICs (in µg/mL and Number of Strains)																			MIC_50_	MIC_90_	ECOFF
		0.0075	0.015	0.03	0.064	0.125	0.25	0.5	1	2	4	8	16	32	64	128	256	512	1024	2048			
A Category	Meropenem	0	**18.46**	**34.87**	**16.41**	**4.62**	**1.54**	**3.08**	**6.15**	**3.59**	**1.03**	**2.05**	**8.21**	0	0	0	0	0	0	0	≤0.03	8	>0.125
	N° Strains		36	68	32	9	3	6	12	7	2	4	16										
	Tigecycline	0	0	0	0	**9.23**	**38.97**	**21.03**	**14.87**	**12.31**	**3.08**	**0.51**	0	0	0	0	0	0	0	0	0.5	2	>1
	N° Strains					18	76	41	29	24	6	1											
	Ceftazidime	0	0	0	0	0	**18.46**	**55.38**	**3.08**	**4.10**	**0.51**	**18.46**	0	0	0	0	0	0	0	0	≤0.5	≥8	>0.5
B Category	N° Strains						36	108	6	8	1	36											
	Colistin	0	0	0	0	0	0	**17.44**	**40.00**	**0.51**	**2.56**	**6.15**	**33.33**	0	0	0	0	0	0	0	≤1	≥16	>2
	N° Strains							34	78	1	5	12	65										
	Nalidixic acid	0	0	0	0	0	0	0	0	**16.41**	**32.82**	**13.85**	**6.67**	**0.51**	**0.51**	**29.23**	0	0	0	0	8	≥128	>16
	N° Strains									32	64	27	13	1	1	57							
	Cefotaxime	0	0	0	0	**18.46**	**57.95**	**3.59**	**0.51**	**1.03**	**18.46**	0	0	0	0	0	0	0	0	0	≤0.25	≥4	>0.25
	N° Strains					36	113	7	1	2	36												
	Ciprofloxacin	**15.90**	**14.87**	**9.23**	**2.05**	**4.62**	**16.92**	**6.67**	**12.31**	**13.33**	**1.03**	**3.08**	0	0	0	0	0	0	0	0	0.25	2	>0.064
	N° Strains	31	29	18	4	9	33	13	24	26	2	6											

The vertical bars indicate the ECOFF value for each antibiotic [17]; The bold numbers indicate the percentage of strains for every MIC value.

**Table 4:** Minimum Inhibitory Concentration (MIC) distributions of antimicrobials against 195 *E. faecalis* strains.

EMA Category	Antimicrobial Agent	Distribution of MICs (in µg/mL and Number of Strains)																			MIC_50_	MIC_90_	ECOFF
		0.0075	0.015	0.03	0.064	0.125	0.25	0.5	1	2	4	8	16	32	64	128	256	512	1024	2048			
A Category	Tigecycline	**2.05**	**0**	**0**	**4.62**	**11.79**	**38.97**	**28.21**	**5.13**	**1.03**	**8.21**	0	0	0	0	0	0	0	0	0	0.25	1	>0.25
	N° Strains	4			9	23	76	55	10	2	16												
	Vancomycin	0	0	0	0	0	0	**54.87**	**10.26**	**13.33**	**10.77**	**9.74**	**0.51**	**0.00**	**0.00**	**0.51**	0	0	0	0	≤1	8	>4
	N° Strains							107	20	26	21	19	1	0	0	1							
	Teicoplanin	0	0	0	0	0	**69.74**	**12.82**	**13.33**	**1.03**	**0.00**	**0.51**	**0.00**	**1.54**	**1.03**	0	0	0	0	0	≤0.5	1	>2
	N° Strains						136	25	26	2	0	1	0	3	2								
	Daptomycin	0	0	0	0	**2.05**	**2.05**	**1.54**	**9.74**	**44.10**	**32.31**	**6.67**	**1.03**	**0.51**	0	0	0	0	0	0	2	4	>4
	N° Strains					4	4	3	19	86	63	13	2	1									
	Linezolid	0	0	0	0	0	**2.05**	**0.00**	**7.69**	**44.10**	**34.36**	**2.05**	**1.03**	**1.03**	**7.69**	0	0	0	0	0	2	8	>4
	N° Strains					0	4	0	15	86	67	4	2	2	15	0	0	0	0	0			
	Quinupristin-dalfopristin	0	0	0	0	0	**3.08**	**0.00**	**3.59**	**12.31**	**18.46**	**27.69**	**21.54**	**7.69**	**5.64**	0	0	0	0	0	8	32	>1
	N° Strains						6		7	24	36	54	42	15	11								
B Category	Ciprofloxacin	0	0	0	**2.05**	**0.51**	**1.03**	**5.64**	**16.92**	**24.10**	**32.82**	**5.64**	**11.28**	0	0	0	0	0	0	0	2	≥16	>4
	N° Strains				4	1	2	11	33	47	64	11	22										

The vertical bars indicate the ECOFF value for each antibiotic [17]; The bold numbers indicate the percentage of strains for every MIC value.

**Table 5:** Multidrug-resistant isolates and resistance profiles observed.

Multirresistance	Microorganism	N° of Strains	%	Resistance Profile	N° of Strains	%
Resistant to 3	*E. coli*	26	13.33	Quinolones Cephalosporins Polymyxins	12	6.15
				Glycylcyclines Cephalosporins Polymyxins	2	1.03
				Glycylcyclines Quinolones Polymyxins	10	5.13
				Carbapenems Quinolones Cephalosporins	2	1.03
	*E. faecalis*	19	9.74	Streptogramins Glycopeptides Quinolones	1	0.51
				Streptogramins Glycylcyclines Quinolones	5	2.56
				Streptogramins Glycylcyclines Glycopeptides	2	1.03
				Streptogramins Glycylcyclines Oxazolidinones	3	1.54
				Streptogramins Glycylcyclines Lipopeptides	3	1.54
				Streptogramins Lipopeptides Quinolones	1	0.51
				Streptogramins Lipopeptides Glycopeptides	3	1.54
				Streptogramins Oxazolidinones Glycopeptides	1	0.51
Resistant to 4	*E. coli*	35	17.95	Glycylcyclines Quinolones Cephalosporins Polymyxins	1	0.51
				Carbapenems Quinolones Cephalosporins Polymyxins	30	15.38
				Carbapenems Glycylcyclines Quinolones Polymyxins	3	1.54
				Carbapenems Glycylcyclines Quinolones Cephalosporins	1	0.51
	*E. faecalis*	13	6.67	Streptogramins Glycylcyclines Lipopeptides Quinolones	1	0.51
				Streptogramins Glycylcyclines Glycopeptides Quinolones	2	1.03
				Streptogramins Glycylcyclines Oxazolidinones Quinolones	7	3.59
				Streptogramins Glycylcyclines Oxazolidinones Glycopeptides	2	1.03
				Streptogramins Lipopeptides Glycopeptides Quinolones	1	0.51
Resistant to 5	*E. coli*	7	3.59	Carbapenems Glycylcyclines Quinolones Cephalosporins Polymyxins	7	3.59
	*E. faecalis*	7	3.59	Streptogramins Glycylcyclines Oxazolidinones Glycopeptides Quinolones	7	3.59

## Abbreviations

AMR: Antimicrobial resistance, ECDC: European Centre for Disease Prevention and Control, ECOFF: epidemiological cut-off value, EFSA: European Food Safety Authority, EMA: European Medicines Agency, MIC: Minimum Inhibitory Concentration.

## References

[1] WHO (2015). Global Action Plan on Antimicrobial Resistance.

[2] Bester L.A., Essack S.Y. (2010). Antibiotic resistance via the food chain: Fact or fiction?. S. Afr. J. Sci..

[3] Hasman H., Hammerum A.M., Hansen F., Hendriksen R.S., Olesen B., Agersø Y., Zankari E., Leekitcharoenphon P., Stegger M., Kaas R.S. (2015). Detection of mcr-1 encoding plasmid-mediated colistin-resistant Escherichia coli isolates from human bloodstream infection and imported chicken meat, Denmark 2015. Eurosurveillance.

[4] Liu Y.Y., Wang Y., Walsh T.R., Yi L.X., Zhang R., Spencer J., Doi Y., Tian G., Dong B., Huang X. (2016). Emergence of plasmid-mediated colistin resistance mechanism MCR-1 in animals and human beings in China: A microbiological and molecular biological study. Lancet Infect. Dis..

[5] Hueston W., Appert J., Denny T., King L., Umber J., Valeri L. (2013). Assessing global adoption of one health approaches. EcoHealth.

[6] Augère-Granier M.L. (2019). The EU Poultry Meat and Egg Sector. Main Features Challenges and Prospects.

[7] EU Commission (1999). Council Directive 99/74/EC of 19 July 1999 laying down minimum standards for the protection of laying hens. Off. J. Eur. Union L.

[8] Ministerio de Agricultura Pesca y Alimentación (2019). Caracterización del Sector Avícola de Puesta en España. Año 2019.

[9] Agunos A., Léger D., Carson C. (2012). Review of antimicrobial therapy of selected bacterial diseases in broiler chickens in Canada. Can. Vet. J..

[10] Landoni M.F., Albarellos G. (2015). The use of antimicrobial agents in broiler chickens. Vet. J..

[11] North American Compendiums Inc. (2003). Compendium of Veterinary Products.

[12] EU Commission Regulation (EC) (2003). No. 1831/2003 of the European Parliament and of the Council of 22 September 2003 on additives for use in animal nutrition. Off. J. Eur. Union L.

[13] EMA (2019). Categorisation of Antibiotics in the European Union. Answer to the Request from the European Commission for Updating the Scientific Advice on the Impact on Public Health and Animal Health of the Use of Antibiotics in Animals.

[14] WHO (2019). Critically Important Antimicrobials for Human Medicine.

[15] Wallmann J. (2006). Monitoring of antimicrobial resistance in pathogenic bacteria from livestock animals. Int. J. Med. Microbiol..

[16] Miranda J.M., Vázquez B.I., Fente C.A., Barros-Velázquez J., Cepeda A., Franco C.M. (2008). Evolution of resistance in poultry intestinal Escherichia coli during three commonly used antimicrobial therapeutic treatments in poultry. Poult. Sci..

[17] EU Commission (2013). Commission implementing decision 2013/652/EU of 12 November 2013 on the monitoring and reporting of antimicrobial resistance in zoonotic and commensal bacteria. Off. J. Eur. Union L.

[18] EFSA, ECDC (2020). The European Union Summary Report on Antimicrobial Resistance in zoonotic and indicator bacteria from humans, animals and food in 2017/2018. EFSA J..

[19] Horrocks S.M., Anderson R.C., Nisbet D.J., Ricke S.C. (2009). Incidence and ecology of Campylobacter jejuni and coli in animals. Anaerobe.

[20] Moore J.E., Corcoran D., Dooley J.S.G., Fanning S., Lucey B., Matsuda M., McDowell D.A., Mégraud F., Millar B.C., O’Mahony R. (2005). Campylobacter. Vet. Res..

[21] Lakhotia R.L., Stephens J.F. (1973). Drug resistance and R factors among enterobacteria isolated from eggs. Poult. Sci..

[22] Mellata M. (2013). Human and avian extraintestinal pathogenic Escherichia coli: Infections, zoonotic risks, and antibiotic resistance trends. Foodborne Pathog. Dis..

[23] Miles T.D., McLaughlin W., Brown P.D. (2006). Antimicrobial resistance of Escherichia coliisolates from broiler chickens and humans. BMC Vet. Res..

[24] Bates J., Jordens Z., Selkon J.B. (1993). Evidence for an animal origin of vancomycin-resistant enterococci. Lancet.

[25] Rivera-Gomis J., Marín P., Otal J., Galecio J.S., Martínez-Conesa C., Cubero M.J. (2021). Resistance patterns to C and D antibiotic categories for veterinary use of Campylobacter spp., Escherichia coli and Enterococcus spp. commensal isolates from laying hen farms in Spain during 2018. Prev. Vet. Med..

[26] EU Commission Regulation (EC) (2003). No 2160/2003 of the European parliament and of the council of 17 November 2003 on the control of Salmonella and other specified food-borne zoonotic agents. Off. J. Eur. Union L.

[27] OIE (2008). *Campylobacter jejuni* and *Campylobacter coli*. OIE Terr. Man..

[28] Linton D., Lawson A.J., Owen R.J., Stanley J. (1997). PCR detection, identification to species level, and fingerprinting of Campylobacter jejuni and Campylobacter coli direct from diarrheic samples. J. Clin. Microbiol..

[29] Nayak R., Stewart T.M., Nawaz M.S. (2005). PCR identification of Campylobacter coli and Campylobacter jejuni by partial sequencing of virulence genes. Mol. Cell. Probes.

[30] Ke D., Picard F.J., Martineau F., Ménard C., Roy P.H., Ouellette M., Bergeron M.G. (1999). Development of a PCR assay for rapid detection of enterococci. J. Clin. Microbiol..

[31] Ge B., Wang F., Sjölund-Karlsson M., McDermott P.F. (2013). Antimicrobial resistance in Campylobacter: Susceptibility testing methods and resistance trends. J. Microbiol. Methods.

[32] EFSA (2020). Manual for reporting on antimicrobial resistance within the framework of Directive 2003/99/EC and Decision 2013/652/EU for information derived from the year 2019. EFSA Support. Publ..

[33] EUCAST (2000). Determination of minimum inhibitory concentrations (MICs) of antibacterial agents by agar dilution. Clin. Microbiol. Infect..

[34] Magiorakos A.P., Srinivasan A., Carey R.B., Carmeli Y., Falagas M.E., Giske C.G., Harbarth S., Hindler J.F., Kahlmeter G., Olsson-Liljequist B. (2012). Multidrug-resistant, extensively drug-resistant and pandrug-resistant bacteria: An international expert proposal for interim standard definitions for acquired resistance. Clin. Microbiol. Infect..

[35] Kahlmeter G., Brown D.F.J., Goldstein F.W., MacGowan A.P., Mouton J.W., Österlund A., Rodloff A., Steinbakk M., Urbaskova P., Vatopoulos A. (2003). European harmonization of MIC breakpoints for antimicrobial susceptibility testing of bacteria. J. Antimicrob. Chemother..

[36] Wales A., Davies R. (2020). Review of hatchery transmission of bacteria with focus on Salmonella, chick pathogens and antimicrobial resistance. Worlds. Poult. Sci. J..

[37] Petersen A., Christensen J.P., Kuhnert P., Bisgaard M., Olsen J.E. (2006). Vertical transmission of a fluoroquinolone-resistant Escherichia coli within an integrated broiler operation. Vet. Microbiol..

[38] Urdahl A.M., Norstrom M., Bergsjø B., Grøntvedt C.A. (2018). The Surveillance Programme for Methicillin Resistant Staphylococcus Aureus in Pigs in Norway 2017. Surveillance Programmes for Terrestrial and Aquatic Animals in Norway. Annual Report 2017.

[39] Aarestrup F.M., Kruse H., Tast E., Hammerum A.M., Jensen L.B. (2000). Associations between the use of antimicrobial agents for growth promotion and the occurrence of resistance among Enterococcus faecium from broilers and pigs in Denmark, Finland, and Norway. Microb. Drug Resist..

[40] Klare I., Badstübner D., Konstabel C., Böhme G., Claus H., Witte W. (1999). Decreased incidence of VanA-type vancomycin-resistant enterococci isolated from poultry meat and from fecal samples of humans in the community after discontinuation of avoparcin usage in animal husbandry. Microb. Drug Resist..

[41] Kühn I., Iversen A., Finn M., Greko C., Burman L.G., Blanch A.R., Vilanova X., Manero A., Taylor H., Caplin J. (2005). Occurrence and relatedness of vancomycin-resistant enterococci in animals, humans, and the environment in different European regions. Appl. Environ. Microbiol..

[42] ECDC (2017). Antimicrobial resistance surveillance in Europe 2015. Annual Report of the European Antimicrobial Resistance Surveillance Network (EARS-Net).

[43] Aznar J., Lepe J.A., Dowzicky M.J. (2012). Antimicrobial susceptibility among E. faecalis and E. faecium from France, Germany, Italy, Spain and the UK (TEST Surveillance Study, 2004–2009). J. Chemother..

[44] O’Dea M., Sahibzada S., Jordan D., Laird T., Lee T., Hewson K., Pang S., Abraham R., Coombs G.W., Harris T. (2019). Genomic, antimicrobial resistance, and public health insights into Enterococcus spp. from Australian chickens. J. Clin. Microbiol..

[45] Dowzicky M., Talbot G.H., Feger C., Prokocimer P., Etienne J., Leclercq R. (2000). Characterization of isolates associated with emerging resistance to quinupristin/dalfopristin (Synercid^®^) during a worldwide clinical program. Diagn. Microbiol. Infect. Dis..

[46] Hershberger E., Donabedian S., Konstantinou K., Zervos M.J., Eliopoulos G.M. (2004). Quinupristin-dalfopristin resistance in gram-positive bacteria: Mechanism of resistance and epidemiology. Clin. Infect. Dis..

[47] Cai Y., Wang R., Liang B., Bai N., Liu Y. (2011). Systematic review and meta-analysis of the effectiveness and safety of tigecycline for treatment of infectious disease. Antimicrob. Agents Chemother..

[48] Van den Bogaard A.E., Hazen M., Hoyer M., Oostenbach P., Stobberingh E.E. (2002). Effects of Flavophospholipol on Resistance in Fecal Escherichia coli and Enterococci of Fattening P. Antimicrob. Agents Chemother..

[49] Donabedian S.M., Perri M.B., Vager D., Hershberger E., Malani P., Simjee S., Chow J., Vergis E.N., Muder R.R., Gay K. (2006). Quinupristin-dalfopristin resistance in Enterococcus faecium isolates from humans, farm animals, and grocery store meat in the United States. J. Clin. Microbiol..

[50] WHO (2004). Joint FAO/OIE/WHO Expert Workshop on Non-Human Antimicrobial Usage and Antimicrobial Resistance: Scientific Assessment: Geneva, 1–5 December 2003.

[51] Hanning I.B., Nutt J.D., Ricke S.C. (2009). Salmonellosis outbreaks in the United States due to fresh produce: Sources and potential intervention measures. Foodborne Pathog. Dis..

[52] Barza M. (2002). Potential mechanisms of increased disease in humans from antimicrobial resistance in food animals. Clin. Infect. Dis..

[53] Helms M., Simonsen J., Olsen K.E.P., Mølbak K. (2005). Adverse health events associated with antimicrobial drug resistance in Campylobacter species: A registry-based cohort study. J. Infect. Dis..

[54] de Been M., Lanza V.F., de Toro M., Scharringa J., Dohmen W., Du Y., Hu J., Lei Y., Li N., Tooming-Klunderud A. (2014). Dissemination of cephalosporin resistance genes between Escherichia coli strains from farm animals and humans by specific plasmid lineages. PLoS Genet..

[55] Lazarus B., Paterson D.L., Mollinger J.L., Rogers B.A. (2015). Do human extraintestinal Escherichia coli infections resistant to expanded-spectrum cephalosporins originate from food-producing animals? A systematic review. Clin. Infect. Dis..

[56] Jakobsen L., Kurbasic A., Skjøt-Rasmussen L., Ejrnæs K., Porsbo L.J., Pedersen K., Jensen L.B., Emborg H.-D., Agersø Y., Olsen K.E.P. (2010). Escherichia coli isolates from broiler chicken meat, broiler chickens, pork, and pigs share phylogroups and antimicrobial resistance with community-dwelling humans and patients with urinary tract infection. Foodborne Pathog. Dis..

[57] PRAN Programme. http://resistenciaantibioticos.es/en.

